# Impaired Taste Perception in Lichen Planus Patients with Tongue Involvement

**DOI:** 10.3290/j.ohpd.b1452911

**Published:** 2021-06-01

**Authors:** Valerie G. A. Suter, Simona Negoias, Hergen Friedrich, Basile N. Landis, Marco-Domenico Caversaccio, Michael M. Bornstein

**Affiliations:** a Senior Lecturer, Department of Oral Surgery and Stomatology, School of Dental Medicine, University of Bern, Bern, Switzerland. Study design, collected and analysed the data, drafted and wrote the manuscript.; b Senior Lecturer, Department for Otorhinolaryngology, Head and Neck Surgery, Basel, University Hospital, Basel, Switzerland; Department of ENT, Head and Neck Surgery, Bern University Hospital, Bern, Switzerland. Study design, collected and analysed the data, drafted and writed the manuscript.; c Senior Lecturer, Department of Otorhinolaryngology, University Children’s Hospital Zurich, Switzerland. Collected the data.; d Associate Professor. Rhinology-Olfactology Unit, Department of Otorhinolaryngology, Head and Neck Surgery, University Hospitals of Geneva, Geneva, Switzerland; Department of ENT, Head and Neck Surgery, Bern University Hospital, Bern, Switzerland. Study design, drafted and wrote the manuscript.; e Professor and Chair, Department for ENT, Head and Neck Surgery, Bern University Hospital, Bern, Switzerland. Drafted and wrote the manuscript.; f Professor and Chair, Department of Oral Health & Medicine, University Center for Dental Medicine Basel UZB, University of Basel, Basel, Switzerland. Study design, collected and analysed the data, drafted and wrote the manuscript.

**Keywords:** mucocutaneous disorders, oral lichen planus, taste/taste physiology, tongue

## Abstract

**Purpose:**

To analyse the taste function in a pool of untreated patients with oral lichen planus (OLP) with tongue lesions (n = 35) and without tongue lesions (n = 36) and to compare it to healthy subjects (n = 36).

**Materials and Methods:**

Firstly, the subjective overall taste ability and impairment of the sensations of ‘sweet’, ‘sour’, ‘salty’ and ‘bitter’ were recorded in all three groups. Secondly, taste function was tested in all included subjects using the standardised ‘Taste Strips’ test.

**Results:**

Data showed a statistically statistically significant difference in overall taste perception between OLP patients with tongue lesions and control subjects (p = 0.027) for the tested taste function. The sensation of ‘sour’ showed the most pronounced difference (p = 0.08). The subjective taste perception and that of individual taste qualities did not differ statistically significantly between the three groups, and the correlation between subjective and objective taste perception was low. There was also a low correlation between taste scores and the presence of lesions on different areas of the tongue.

**Conclusion:**

For patients with OLP experiencing a loss in appetite, a formal taste examination and subsequent counselling should be considered.

Oral lichen planus (OLP) is a chronic inflammatory and immune-mediated disease affecting the oral mucosa.^1,3,18^ Population-based studies have reported a prevalence between 0.06% and 3.2% with a predominance at over 50 years of age and in females.^5,16,21^ OLP is classified into 6 different clinical types: reticular (white striae), plaque-like (white plaques), papular (white papules), atrophic (erythema), erosive (erosions and/or ulcers), and bullous (blisters).^1,11^ Typically, different types are found simultaneously in various sites of the mouth. The hyperkeratotic white types are usually asymptomatic. When atrophy, erosions and ulcers are present, patients often report pain and a burning sensation.^2,4^ The presence of white striae and more or less bilateral involvement are both mandatory for the diagnosis of OLP. This must be further confirmed by typical histopathological characteristics.^7,27,28^

The buccal mucosa is the most affected site, followed by the tongue and the gingiva.^2,3,5,16,25^ Involvement of the dorsum of the tongue presents as atrophy of the papillary structure, erosion/ulceration and/or mucosal thickening, with white striae and plaques in the anterior two-thirds.

Perception of taste is induced by the activation of taste receptors found on the taste buds. Within the oral cavity, taste buds have the highest density on the tongue. Each taste bud can contain up to 100 neuroepithelial cells with receptors able to recognise all of the five human basic tastes ‘sweet’, ‘sour’, ‘salty’, ‘bitter’ and ‘umami’.^6,14^ The front, rear and sides of the tongue are more sensitive to gustatory stimuli.^32^ A decline in taste sensation can lead to a decrease in the quality of life and depression.^14^ Furthermore, restricted taste sensation results in an impaired selection of food variety, a decrease in the intake of fruit and vegetables, and consumption of more salty and sweet foods. This can be associated with vitamin deficiency, weight gain, type II diabetes and increased caries activity.^15^ Factors known to influence taste sensation are systemic diseases, medication, neurological diseases – including peripheral nerve damage – the patient’s cognitive state, viral infections like COVID-19, and idiopathic reasons.^8,16^ Quantitative taste testing can be performed on the dorsum of the tongue with the standardised ‘Taste Strips’ test (Burghart; Wedel, Germany). This is a validated method to test four (‘sweet’, ‘sour’, ‘salty’, ‘bitter’) of the five basic taste sensations.^14,20^ On the other hand, to detect qualitative taste disorders, questionnaires are required.^13,14,26^

The aim of the present case-control study was to test the taste function in a pool of patients with OLP compared to a healthy control group and to find any correlation with the region of the tongue affected in OLP patients. Thus, the hypothesis of the study was that an altered objective taste sensation in patients with oral lichen planus and lesions on the dorsum of the tongue would be present when compared to OLP patients without tongue lesions (positive controls) and also to healthy subjects (negative controls). Furthermore, the subjective taste perception of all subjects was evaluated and correlated to the tested taste function.

## Materials and Methods

### Study Sample, Inclusion and Exclusion Criteria

The study protocol was approved by the local ethics committee of the canton of Bern, Switzerland (approval number 166/13). The study was conducted according to the guidelines of the World Medical Association Declaration of Helsinki.^31^ The reporting of this case-control study followed the STROBE guidelines for the reporting of observational studies.^29^

Patients referred to us with suspected OLP or attending an annual follow-up at the University of Bern’s Department of Oral Surgery and Stomatology were initially eligible. To be included, the clinical and histopathological features of OLP as initially proposed by the WHO Collaborating Center for Oral Precancerous Lesions had to be present.^7,19,27,28^

For histopathologic examination by an experienced pathologist, an intraoral area with representative lesions was selected, and an incisional biopsy was performed (MB or VS). Patients with ongoing treatment for OLP were excluded. Further exclusion criteria were olfaction disorders; acute or chronic rhinosinusitis; head, neck and throat diseases; previous ENT surgery; previous radiochemotherapy; and medications or medical conditions (i.e. pregnancy, diabetes mellitus) known to induce taste disturbances. All respective ENT examinations were performed by HF or SN.

Control subjects recruited from the patient pool of the Department of Oral Surgery and Stomatology were examined by MB or VS and their eligibility to participate was confirmed based on an inconspicuous oral mucosa without any oral diseases or variation (e.g. geographic tongue). The other abovementioned exclusion criteria for the test group also applied to control subjects. Written informed consent was obtained from all included subjects.

The sample size was chosen according to a power analysis based on an age-related difference of points in the taste perception score between a person with impaired taste and one without. With a statistical significance level set at 0.05 and an assumed median taste score of 21.4, the standard deviation was 6.6 points. Sample size calculation resulted in 30 patients per group to obtain a power of 80%. The study was planned to compare subjective and objective taste in three different groups of patients: patients with OLP and tongue lesions (group 1); patients with OLP, but without tongue lesions (group 2); and a control group including patients without OLP and without tongue lesions (group 3). The subjects were recruited between November 2013 and September 2016.

### Clinical Features

**Fig 1:**
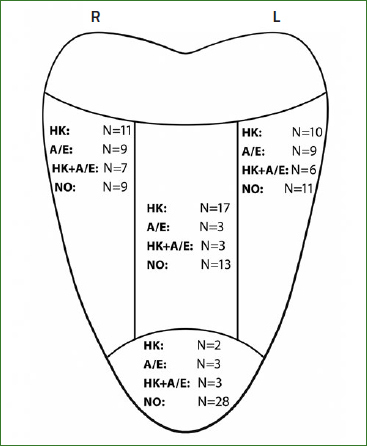
Division of the dorsum of the tongue into five sections (posterior, left, middle, right and tip), and the distribution of the type of lesions observed in the four anterior sections for the 35 patients included in group 1. HK: hyperkeratotic reticular and/or plaque-like only; A/E: atrophic and/or erosive only; HK+A/E: mixed hyperkeratotic (reticular and/or plaque-like) and atrophic/erosive. NO: no lesions.

The clinical features of OLP were recorded.^1,11^ To evaluate the dorsum of the tongue, the reticular, papular and plaque-like types were grouped into a hyperkeratotic phenotype, and the atrophic (depapilated), erosive/ulcerative and rare bullous types into an atrophic-erosive phenotype. The dorsum of the tongue was divided into five sections (Fig 1). The area posterior to the vallate papillae was not graded, and the four anterior sections (left, middle, right and tip) were classified according to the mucosal phenotype as 1) no lesion, 2) hyperkeratotic only, 3) atrophic-erosive only, 4) mixed (hyperkeratotic and atrophic-erosive).

### Patient’s Subjective Pain and Taste Assessment

For the first study-related assessment, the patients rated intraoral pain due to OLP on a visual analogue scale (VAS, ranging from 0 to 100 mm). Second, all subjects were asked to rate their subjective taste perception using the VAS (ranging from 0 to 100 mm; 0: my taste function is very poor; 100: I have extraordinarily good taste function). Next, they rated their level of daily life impairment caused by taste problems (VAS ranging from 0 to 100 mm; 0: I am not impaired by taste problems: 100: I am extremely impaired by my taste problems). For the qualitative assessment, a written questionnaire was also completed by the subjects. They answered the question ‘Does food taste different?’ by choosing one of the four options ‘never, seldom, often, always’, both in general and with regard to the four taste qualities ‘sweet’, ‘sour’, ‘salty’ and ‘bitter’. They also answered with ‘yes’ or ‘no’ this question: ‘Have you noticed any impairment of taste since having a mucosal disease?’

### Taste Testing

A validated test containing ‘Taste Strips’ was used for taste testing (Taste Strips, Burghart; Wedel, Germany).^20^ Subjects were asked to stick out their tongue and to keep it in a resting position during application of the Taste Strip. The tongue dorsum of each subject was then tested on the right and left sides for each of the four taste qualities and in four different concentrations: ‘sweet’: 0.4, 0.2, 0.1, 0.05 g/ml sucrose; ‘sour’: 0.3, 0.165, 0.09, 0.05 g/ml citric acid; ‘salty’: 0.25, 0.1, 0.04, 0.016 g/ml sodium chloride; ‘bitter’: 0.006, 0.0024, 0.0009, 0.0004 g/ml quinine hydrochloride. To avoid any dispersion or dilution of the agent, subjects were asked not to talk during the procedure. Cards with the four different taste qualities written on them were accessible to the subjects to point towards the correct answer in a ‘forced-choice procedure’, meaning that they were obliged to select one of the taste qualities. The different tastes varied in order, but otherwise all subjects were tested identically, and a short break without rinsing the mouth was always maintained between each strip application. For each correctly identified taste, one point was given, resulting in values between 0 and 4 for each taste quality and side of the tongue. Total scores were calculated for the right and left sides of the tongue.

### Statistical Analysis

First, the two patient groups and the control group were matched in order to have three comparable groups with respect to age and sex. In a second step, descriptive analyses were performed separately for each group as well as for the three groups together. In a third step, an overall comparison of objective and subjective taste perception of the three groups was performed using the Kruskal-Wallis test. For pairwise comparison of the groups, Mann-Whitney-Wilcoxon tests were performed. Tests measuring a potential correlation between objective and subjective taste perception were performed. Furthermore, correlation tests between taste perception and type, localisation and number of tongue sections affected were performed for group 1. Spearman’s rank correlation coefficient (ρ) was caluculated to determine correlations and presented as estimates together with 95% confidence intervals. Nonparametric methods were chosen due to sample size. All p-values were corrected for multiple testing. p-values < 0.05 were considered statistically significant. All results were obtained with R, 3.3.3 (R Core Team 2013. R Foundation for Statistical Computing; Vienna, Austria).

## Results

**Fig 2:**
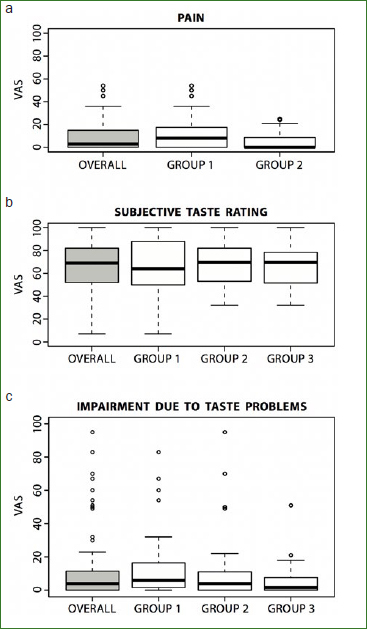
Box-plot representation of pain sensations (a) for all OLP patients and for groups 1 and 2 separately using a VAS (1-100 mm). Box-plot representation of subjective taste perception (b) and subjective impairment due to taste problems (c) evaluated using a VAS (1-100 mm) for all subjects pooled and for all three groups separately. Group 1: patients with OLP and tongue lesions; group 2: patients with OLP without tongue lesions; group 3: control subjects.

Overall, 71 patients with clinically and histopathologically confirmed OLP and 36 healthy subjects were included in the study. The female:male ratio of the control group was 2:1. The female:male ratio was similar for OLP patients (2.4:1). Half of the OLP patients did not present with a tongue lesion (n = 36, group 2). Figure 1 shows the types of lesions found on the dorsum of the tongues of the 35 patients in group 1. Demographic data and smoking habits of all subjects are shown in Table 1. The mean pain sensation of patients with OLP was 8.75 (VAS 1–100), and there was no statistically significant difference between OLP patients with and without tongue lesions (Fig 2a).

**Table 1:** Demographic data of the 107 subjects included (age, gender and smoking habits)

	Overall	Group 1 Subjects with OLP and tongue lesions	Group 2 Subjects with OLP and without tongue lesions	Group 3 Control subjects (no mucosal lesion)
**Subjects (n)**	107	35	36	36
**Age (years)**				
Mean	60.03	61.66	58.75	59.72
Median (p25, p75)	59.00 (52.50, 67.00)	59.00 (54.00, 69.00)	58.50 (52.00, 63.25)	60.50 (51.00, 66.50)
Minimum	27.00	27.00	33.00	39.00
Maximum	88.00	88.00	82.00	87.00
**Gender (n)**				
Female, n (%)	74 (69%)	24 (69%)	26 (72%)	24 (67%)
Male, n (%)	33 (31%)	11 (31%)	10 (28%)	12 (33%)
**Smoking**				
yes, n (%)	9 (8%)	3 (9%)	3 (8%)	3 (8%)
no, n (%)	98 (92%)	32 (91%)	33 (92%)	33 (92%)

p25: 25th percentile; p75: 75th percentile; OLP: oral lichen planus.

Subjective taste perception was similar for all groups (Fig 2b). Regarding their level of daily life impairment caused by taste problems, OLP patients with tongue lesions were subjectively not more affected than patients from the two other groups (Fig 2c). Similarly, there was no difference between the groups regarding the subjective qualitative taste sensations (‘sweet’, ‘salty’, ‘sour’, ‘bitter’). Taste impairment since having OLP was reported by eight patients (11.2%, 6 patients with and 2 without tongue involvement, Table 2).

**Table 2:** Gender, age, localisation of tongue lesions and objective taste test scores (by Taste Strips) of the eight patients (#1 – #8) with subjective impairment of taste since affected by oral lichen planus

	Gender	Age	Localisation and type of lesions on the tongue (0: no lesion; 1: hyperkeratotic; 2: atrophic-erosive; 3: mixed)		Subjective loss of taste VAS 0 (not impaired) to 100 (extremely impaired)	Taste strip test scores				
						Overall 0-32	Sweet 0-8	Sour 0-8	Salty 0-8	Bitter 0-8
#1	m	53	lateral right	3	32	14	8	1	4	1
			middle	0						
			lateral left	0						
			tip	0						
#2	m	56	lateral right	2	69	23	7	4	5	7
			middle	2						
			lateral left	2						
			tip	0						
#3	f	88	lateral right	2	54	18	3	4	3	8
			middle	2						
			lateral left	2						
			tip	0						
#4	f	56	lateral right	3	83	13	4	3	0	6
			middle	0						
			lateral left	2						
			tip	0						
#5	f	81	lateral right	2	60	25	6	7	6	6
			middle	0						
			lateral left	2						
			tip	0						
#6	f	54	lateral right	0	67	23	7	4	7	5
			middle	0						
			lateral left	3						
			tip	1						
#7	f	50	lateral right	0	70	21	8	4	4	5
			middle	0						
			lateral left	0						
			tip	0						
#8	m	33	lateral right	0	50	25	7	6	7	5
			middle	0						
			lateral left	0						
			tip	0						

**Fig 3:**
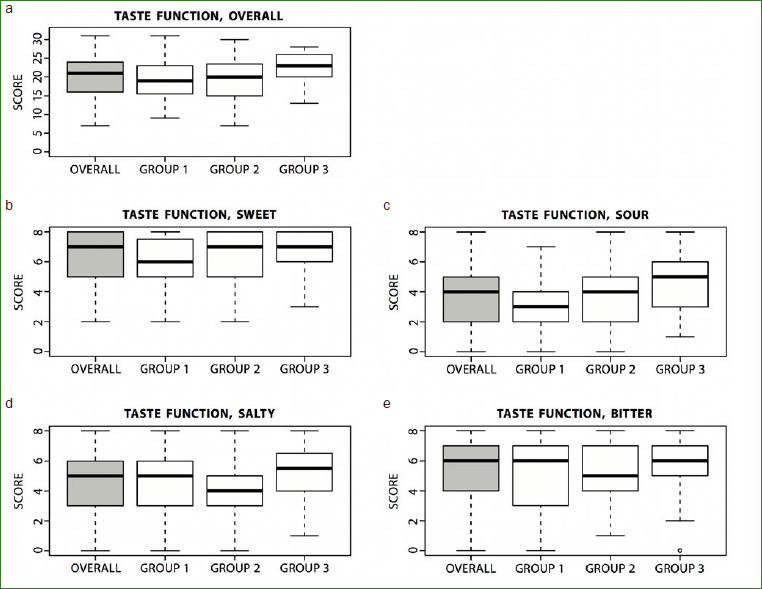
Box-plot representing the taste function measured by Taste Strips overall (a; score 0-32) and for the taste of ‘sweet’ (b; score 0-8), ‘sour’ (c; score 0-8), ‘salty’ (d; score 0-8), and ‘bitter’ (e; score 0-8) in the three groups. Group 1: patients with OLP and tongue lesions; group 2: patients with OLP without tongue lesions; group 3: control subjects.

The Kruskal-Wallis tests for overall comparisons of taste perception between the three groups did not demonstrate statistically significant results. A statistically significant difference (p = 0.027) for taste function was found in pairwise comparisons using Mann-Whitney-Wilcoxon tests between patients with OLP affected by tongue lesions (group 1) and control subjects (group 3) (Fig 3). When analysed further for taste qualities, the sensation ‘sour’ showed the most pronounced difference (p = 0.08). For patients with OLP not affected by tongue lesions, there was a statistically significant difference in the taste sensation ‘salty’ compared to control subjects (p = 0.029). For both taste qualities, the control subjects performed better than did the OLP patients.

The correlation between subjective and objective taste perception was low (ρ between -0.3 to 0.3) overall and for most of the different tastes in all three groups. A medium correlation between subjective and objective taste perception was found for the overall taste impairment in group 2 (ρ = 0.423, CI [0.122, 0.672]), and for the perception of ‘sweet’ in group 1 (ρ = -0.428, CI [-0.611, -0.185]). In group 1, there was a low correlation (ρ = -0.3 to 0.3) between taste scores and the presence of lesions in the different areas of the tongue (Fig 1). A low correlation (ρ = -0.3 to 0.3) was also found between the type of lesion, the number of affected lesions and taste scores.

## Discussion

The present study showed a lower overall measured taste perception in patients affected by OLP with tongue lesions compared to healthy subjects, and this was most pronounced for the taste ‘sour’. Patients with OLP without tongue lesions had a lower perception of ‘salty’ than healthy subjects. Subjectively, however, taste perception in patients with OLP was not more disturbed than was the case among healthy subjects. The ‘Taste Strips’ test is a straightforward test for the measurement of taste function. It has been validated on healthy subjects and used in various studies assessing taste function.^20,22,24,30^ To the best of our knowledge, this is the first sufficiently powered study testing gustatory function in patients with OLP with and without tongue lesions. Our own group previously published preliminary data from an OLP group with tongue lesions.^26^ Increasing the study population confirmed that overall taste perception was reduced in the group of OLP patients with tongue lesions.

The overall lower taste perception in patients affected by OLP with tongue lesions confirmed the hypothesis of the study. The diminished sensation could be explained by the lower density of taste buds and papillae on the dorsum of the tongue affected by atrophy, erosion, ulceration or hyperkeratosis in conjunction with OLP. However, the type of lesion did not seem to be the determining factor, as in the present study a subgroup analysis showed only a low correlation between lesion type and taste perception scores. Nevertheless, it is important to consider that taste buds are also found on the palate, oropharynx, larynx, upper oesophagus and intestine, but it is unlikely that the latter contribute to the perception and discrimination of taste.^6^

Taste function also varies in healthy subjects and according to ethnocultural background, age and gender of patients. It has been observed that people living in the Mediterranean region have lower taste threshold scores than do Germans or Austrians,^24^ women have better taste perception than men,^20^ and that the taste function decreases with age.^20,23,33^ A Japanese study showed reduced taste perception in the elderly for all taste qualities with the exception of ‘sweet’ in comparison to young adults.^33^ In general, the diminished sensation of taste in the elderly is also attributed to a lower quantity of taste buds. In the present study, the subjects of all three groups were of a similar age; thus, age should not have been a confounding factor. The study population was from the same ethnocultural environment and gender distribution was equal between the groups. The fact that we selected our own control group of patients with inconspicuous oral mucosa, who were tested by the same investigator, is a further advantage of the present study, in contrast to the comparison of results of a population tested elsewhere.

In the present study, it was the ‘sour’ taste that was predominantly reduced in the OLP patients with tongue lesions. A study of an Israeli population showed that persons with symptomatic OLP avoided eating citrus fruits and tomatoes, as well as hard or spicy foods.^12^ Also, a statistically significant association was found between food preferences and lesion locations, with a higher prevalence of tongue lesions in patients avoiding citrus fruits. A possible explanation for the lower ‘sour’ scores of OLP subjects with tongue lesions in the present study is that they were less trained in recognising ‘sour’ due to avoiding it. It has also been shown that there is a better performance for citric acid at the tip of the tongue.^14,23^ In the present study however, no correlation could be found between the lower score of ‘sour’ and the location of lesions in OLP patients.

The inclusion of a group of patients with OLP but without tongue lesions seems to confirm that the taste alteration is more pronounced when the tongue is affected, as overall taste perception did not differ between healthy subjects and the group of OLP patients without tongue lesions. However, Mann-Whitney-Wilcoxon analysis showed an altered perception of ‘salty’ in the group of OLP patients without tongue lesions compared to healthy subjects. Besides the possibility that persons with OLP without tongue lesions have a reduced salty perception, it is also possible that people of this group had a lower threshold of recognition. This has been described in patients affected by heart disease and those on a low-salt diet. It has been stated that a general reduction in salt intake decreases its threshold recognition.^9^ These specific health and dietary factors – which could have been a confounding factor in this elderly population – were not considered in this study. In the literature, a possible association between a higher risk for cardiovascular disease and OLP, particularly in patients with erosive-atrophic lesions, probably due to the chronic inflammatory state, has been described.^10^ However, there is no description in the literature of a known association between OLP and the tendency to avoid salt in food.

Treatment of OLP is recommended for erosive, ulcerative and chronic inflammatory lesions. Usually, these patients also request treatment due to pain associated with these lichen manifestations. Some patients may not complain about pain, but mention an unspecified soreness in their mouth. Thus, pain, soreness and the type of lesions present are considered as determining factors for choosing the treatment modality for OLP, and not the taste function. It would however be interesting to test patient’s taste scores over time with and without treatments to see if there is any beneficial effect.

## Conclusion

Patients affected by OLP and with tongue lesions can have an impaired overall taste perception, including a specific impairment of the perception of ‘sour’ taste. Interestingly, OLP patients may not complain of taste impairment as they are often not subjectively disturbed. Thus, in daily clinical practice, there is no necessity for taste testing in OLP patients on a routine basis. However, if OLP patients exhibit reduced appetite, a taste perception test may be indicated to identify a possible cause.
